# Supplementary material for: Risk of circulatory diseases associated with proton-pump inhibitors: a retrospective cohort study using electronic medical records in Thailand
Source: PeerJ. 2024 Feb 15;12:e16892. doi: 10.7717/peerj.16892 (PMC10874595; doi:10.7717/peerj.16892)
Supplement: Supplemental Information 1 [file peerj-12-16892-s001.docx]

**Supplementary**

**Data Description**

This section describes how to define exposure and outcome variables in the study. The exposures were divided into 3 classes of gastric acid suppressants—Proton Pump Inhibitors, Histamine 2 receptor antagonist, and miscellaneous drugs.

**Table 1S** Gastric suppressants’ agents using in the study

| **HOSPITAL**  **DRUG_CODE** | **Brand_NAME** | **GENERIC_NAME** | **Data Coding** |
| --- | --- | --- | --- |
| 339 | Cimetidine 200 mg Tab | CIMETIDINE | H2RA |
| 539 | Tagamet 800 mg Tab (R) | CIMETIDINE | H2RA |
| 589 | Zantac 150 mg Tab (R) | RANITIDINE | H2RA |
| 685 | Cimetidine 200 mg/2ml injection | CIMETIDINE | H2RA |
| 819 | Zantac 300 mg Tab (R) | RANITIDINE | H2RA |
| 895 | Ranitidine 300mg Tab | RANITIDINE | H2RA |
| 934 | Axid 300 mg Cap | NIZATIDINE | H2RA |
| 961 | Axid 150 mg Cap | NIZATIDINE | H2RA |
| 1055 | Cimetidine 400 mg Tab | CIMETIDINE | H2RA |
| 851 | Ranitidine 150 mg Tab | RANITIDINE | H2RA |
| 1023 | Ranitidine inj 50 mg/2 ml | RANITIDINE | H2RA |
| 2011 | Aluminium Hydroxide 240ML | AL(OH)3 | OTHER |
| 357 | De-Nol 120 mg | BISMUTH SUBCITRATE | OTHER |
| 393 | Gelusil MPS Tab | AL(OH)3+MG(OH)3+SIMETHICONE | OTHER |
| 9012 | Antacid Susp 240 ml | AL(OH)3+MG(OH)3 | OTHER |
| 9013 | Alum gel (Alumin)  240 ml | AL(OH)3 | OTHER |
| 560 | Ulsanic 1 gm Tab | SUCRALFATE | OTHER |
| 1156 | Ulcefate 1 gm/5 ml 60ml | SUCRALFATE | OTHER |
| 2799 | Gaviscon SAC Suspension | SOD ALGINATE, NAHCO3, CACO3 | OTHER |
| 275 | Aluminium hydroxide Tab | AL(OH)3 | OTHER |
| 392 | Aluminium+Magnesium Tab | AL(OH)3+MG(OH)3 | OTHER |
| 1595 | Aluminium+Magnesium 240ml | AL(OH)3+MG(OH)3 | OTHER |
| 2676 | Pariet 20 mg Tab @ | RABEPRAZOLE SODIUM | PPI |
| 1569 | Nexium 20 mg | ESOMEPRAZOLE | PPI |
| 918 | Losec 20 mg Mups Cap (R) | OMEPRAZOLE | PPI |
| 983 | Losec 40 mg Inj (R) | OMEPRAZOLE | PPI |
| 2747 | Axiago 20 mg Tab | ESOMEPRAZOLE | PPI |
| 2036 | Prevacid FDT 15mg | LANSOPRAZOLE | PPI |
| 2072 | Prevacid FDT 30mg | LANSOPRAZOLE | PPI |
| 1162 | Omeprazole 20 mg Cap | OMEPRAZOLE | PPI |
| 1733 | Omeprazole(40mg) Inj | OMEPRAZOLE | PPI |
| 9727 | Omepraz.TF 2mg/ml Sus60mI | OMEPRAZOLE | PPI |
| 1697 | Pantoval 40mg Inj | PANTOPRAZOLE SODIUM | PPI |
| 2646 | Dexilant DR 30 mg Cap | DEXLANSOPRAZOLE | PPI |
| 2647 | Dexilant DR 60 mg Cap | DEXLANSOPRAZOLE | PPI |

Primary outcomes using ICD-10-CM are defined as seen on the below tables.

**Table 2S** Study outcomes using ICD-10-CM.*

| **No.** | **Primary outcome** | **Disease** | **ICD-10-CM** |
| --- | --- | --- | --- |
| 1 | Cardio Vascular Disease | Heart Failure | I42, I42.0-I42.9, I43, I50, I50.0-I50.9, I11, I11.0 |
|  |  | Cardiac arrythmia | I48, I48.0-I48.9, I49, I49.0-I49.5, I49.8, I49.9 |
|  |  | Ischemic Heart Disease | I20, I20.0, I20.1, I20.8, I20.9, I21, I21.0-I21.4, I21.9, I21.A, I21.A1, I21.A9, I22, I22.0-I22.2, I22.8, I22.9, I23, I23.0-I23.8, I24, I24.0, I24.1, I24.8, I24.9, I25, I25.0-I25.9 |
| 2 | Ischemic Strokes | Cerebral infarction | I63, I63.0-I63.6, I63.8, I63.9, I64 |
| 3 | Peripheral Vascular Disease |  | I70, I70.0-I70.9 |

*The accuracy of International Statistical Classification of Disease and Related Health Problems, 10^th^ Revision (ICD-10) coding system in identifying comorbidities, including cardiovascular disease, pheripheral vascular disease, and cerebro vascular diseases (including ischemic strokes) using data from a Thai university hospital administrative database were published. (Rattanaumpawan, Wongkamhla, & Thamlikitkul, 2016) We have not collected accuracy of ICD-10 codes at the study site in this study

**
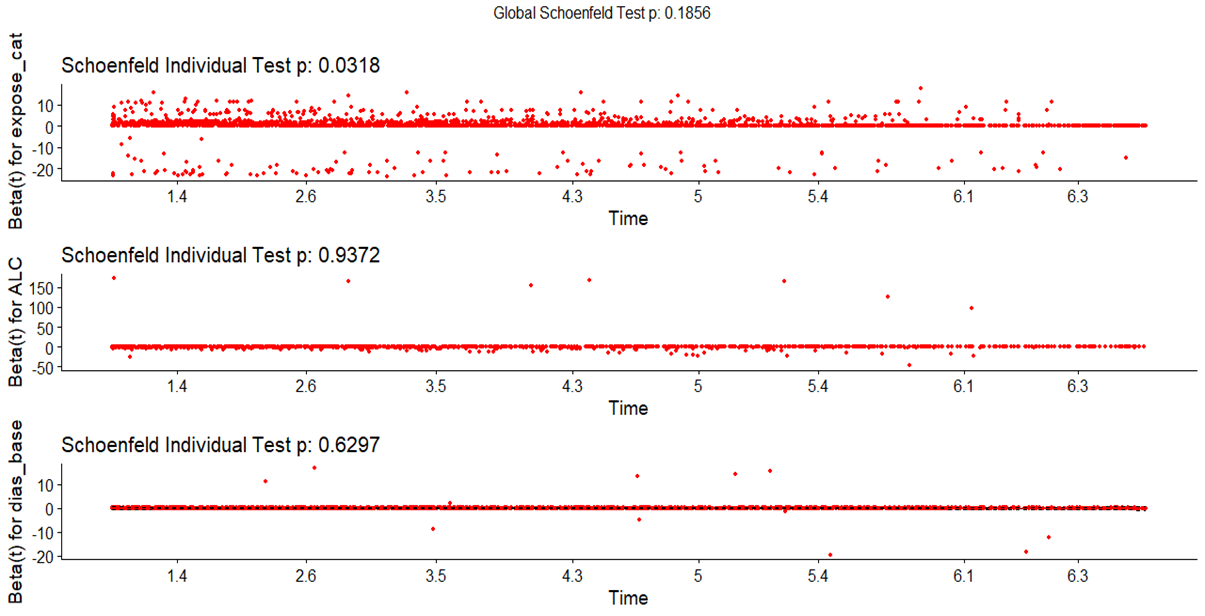
**

**Figure 1S** Test of proportional hazard assumption with global Schoenfeld residual test (p-value>0.05)

**Table 3S** Baseline demographic and health characteristics at index time between PSM and excluded participants after 1:1 matching

|  |  | **Total** | **PSM** | **Excluded** | **p-value*** |
| --- | --- | --- | --- | --- | --- |
| **Number of participants (%)** |  | **59,322 (100.00)** | **7,856 (100.00)** | **51,466 (100.00)** |  |
| **Age group** |  |  |  |  | **0.442** |
|  | ≤60 (%) | 39,871 (67.21) | 5,626 (71.61) | 34,245 (66.54) |  |
|  | >60 (%) | 19,451 (32.80) | 2,230 (28.39) | 17,221 (33.46) |  |
| **Sex** |  |  |  |  | **0.657** |
|  | Female (%) | 37,810 (63.74) | 5,187 (66.03) | 32,623 (63.387) |  |
|  | Male (%) | 21,512 (36.26) | 2,669 (33.97) | 18,843 (36.61) |  |
| **Index Years (Fiscal years)** |  |  |  |  | **0.068** |
|  | 2011(%) | 19,532 (32.92) | 1,399 (17.81) | 18,133 (35.23) |  |
|  | 2012(%) | 12,059 (20.33) | 1,357 (17.27) | 10,792 (20.97) |  |
|  | 2013(%) | 9,101 (15.34) | 2,162 (27.52) | 6,939 (13.48) |  |
|  | 2014(%) | 6,148 (10.36) | 1,055 (13.43) | 5,093 (9.90) |  |
|  | 2015(%) | 4,948 (8.34) | 881 (11.21) | 4,067 (7.90) |  |
|  | 2016(%) | 4,186 (7.06) | 592 (7.53) | 3,594 (6.98) |  |
|  | 2017(%) | 3,348 (5.64) | 500 (6.36) | 2,848 (5.53) |  |
| **Charlson Co-morbidity Index (IQR)** |  | 0 (0-1) | 0 (0-1) | 0 (0-1) | **0.100** |
| **Diagnoses** | Diseases of esophagus, stomach, and duodenum (K20-K31) (%) | 67 (0.11) | 10 (0.13) | 57 (0.11) | **1.000** |
|  | Malignant neoplasms of digestive organs (C15-C26) (%) | 458 (0.77) | 105 (1.34) | 353 (0.69) | **0.820** |
|  | Infectious gastroenteritis (B96.81, A09, A04.7) (%) | 440 (0.74) | 61 (0.78) | 379 (0.74) | **0.750** |
|  | Hypertension (I10-I16) (%) | 10,750 (18.12) | 1,328 (16.90) | 9,422 (18.31) | **0.852** |
|  | Diabetes Mellitus (E10-E14) (%) | 5,421 (9.14) | 679 (8.64) | 4,742 (9.21) | **0.106** |
|  | Hyperlipidemia (E78.XX) (%) | 11,883 (20.03) | 1,343 (17.10) | 10,540 (20.48) | **0.562** |
|  | Renal Disease (N02-N08, N11, N12, N14, N18, N19, N26, N158, N159, N160, N162, N163, N164, N168, E102, E112, E132, E142) (%) | 2,422 (4.08) | 361 (4.60) | 2,061 (4.00) | **0.733** |
|  | Rheumatoid Arthritis (M05, M06, M32, M33, M34, M353) (%) | 1,220 (2.06) | 113 (1.44) | 1,107 (2.15) | **0.561** |
|  | Hypomagnesemia (E83.42) (%) | 172 (0.29) | 41 (0.52) | 131 (0.26) | **0.759** |
|  | Alcohol abuse (E244, E52, F1, G312, G621, G721, I426, K292, K70, K860, L278A, N979, N980, O354, T51, Z714, Z721) (%) | 333 (0.56) | 78 (0.99) | 255 (0.49) | **0.684** |
|  | Smoking abuse (F17, O99.33, T65.2, Z87.891) (%) | 1 (0.002) | 1 (0.01) | 0 (0.00) | **1.000** |
| **Co-Prescription** | Angiotensin-Converting Enzyme Inhibitors (ACEIs) (%) | 3,427 (5.78) | 471 (5.99) | 2,956 (5.74) | **0.373** |
|  | Angiotensin Type II Receptor Antagonist (AIIA) (%) | 1,408 (2.37) | 163 (2.07) | 1,245 (2.42) | **1.000** |
|  | Acetylsalicylic acid (aspirin) (%) | 5,555 (9.36) | 714 (9.09) | 4,841 (9.41) | **0.368** |
|  | Non-steroidal Anti-inflammatory drugs (NSAIDs) (%) | 33,204 (55.97) | 1,953 (24.86) | 31,251 (60.722) | **0.000**** |
|  | Steroids (%) | 6,828 (11.51) | 1,898 (24.16) | 4,930 (9.58) | **0.008**** |
|  | Diuretics (%) | 4,738 (7.99) | 590 (7.51) | 4,148 (8.06) | **0.804** |
|  | Statins (%) | 8,696 (14.66) | 1,093 (13.91) | 7,603 (14.77) | **0.805** |
|  | Digoxin (%) | 482 (0.81) | 57 (0.73) | 425 (0.83) | **1.000** |
|  | Aminoglycosides (%) | 385 (0.65) | 69 (0.88) | 316 (0.61) | **1.000** |
| **Number of hospital visits (IQR)** |  | 3 (1-7) | 4 (2-9) | 3 (1-7) |  |
| **Blood Pressure**** |  |  |  |  |  |
|  | Systolic (SD) | 129.02 (19.83) | 126.67 (19.55) | 129.37 (19.84) | **0.000**** |
|  | Diastolic (SD) | 76.69 (14.25) | 75.99 (14.30) | 76.79 (14.24) | **0.000**** |
| **BMI [kg/m2] (SD)** | Number of participants (%) = 9,123 (15.38) | 24.98 (4.76) | 25.63 (5.56) | 24.76 (4.44) | **0.000**** |

**Notes:** * Chi-square test for categorical data using percentages and independent t-test for continuous data, **significant level < 0.05

**Propensity Score Matching.**

We applied propensity score matching (PSM) to estimate the average treatment effect in the treated (ATT) on exposures on eligible participants who received it accounting for confounding by the included baseline covariates. We used 1:1 nearest neighbor propensity score matching without replacement with caliper = 0.10; propensity score estimated using logistic regression of the treatment on the covariates with *MatchIt* package in R program. Since the imbalance of covariates before matching regarding Love plot (Figure 1B), we optimally tried to fit the estimated model, particularly to mitigate the huge absolute standardized differences (ASDs) of covariates as much as possible. The ASDs were computed as the mean difference divided by a standardization factor computed in the unmatched sample. An absolute ASDs less than 0.1 was acceptable value for potentially prognostically important variables appearing in several recommendations in the literature(Zhao et al., 2021).


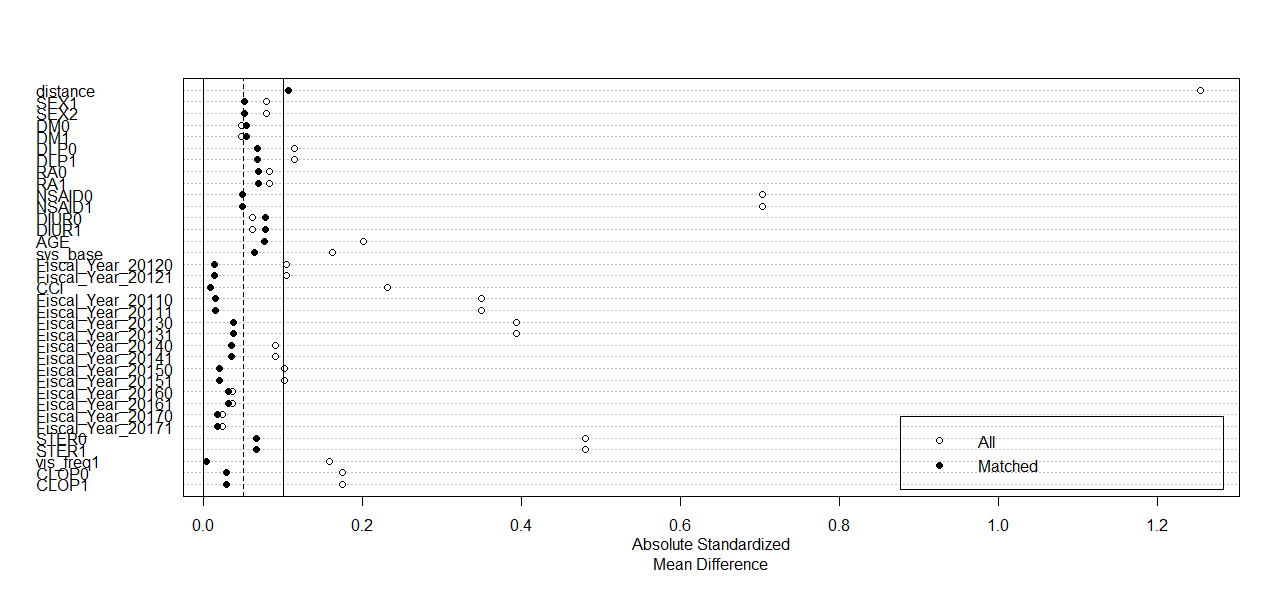


**Figure 2S** Love plot of ASDs among baseline covariates Before PSM (All) vs After PSM (Matched) with selective covariates.

To trade-off between predictive value and matched-pairs’ sample size, we prioritize to mitigate the confounding effects of some covariates—sex, age, CCI, diabetes, dyslipidemia, rheumatoid arthritis, NSAIDs, diuretics, steroids, clopidogrel, index years, number hospital visits, and, systolic blood pressure, while, using receiver operating characteristic curve (ROC) with area under the curve (AUC) more than 70% to be the criterion of selected PSM model.

After matching, the matching procedure left 5 control and 51,461 treated units unmatched. Selected covariates showed the improved balance with ASDs in PSM estimation, (Figure 2S), while, its AUC is by 78.31%. (Figure 3S)


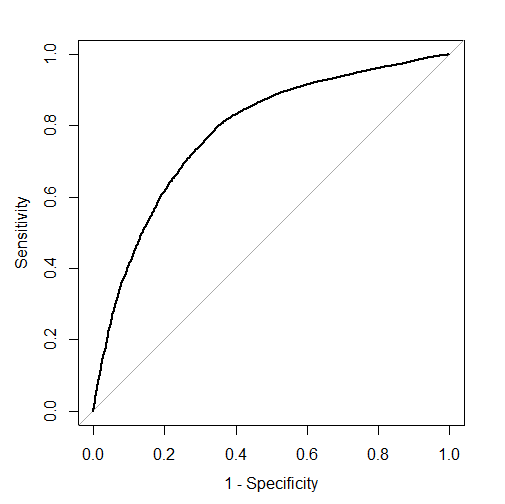


**Figure 3S** Receiver operating characteristic curve with area under the curve of the propensity score model using selective baseline covariates.

**TABLE 4S** Multivariable Cox Proportional Hazards: Overall survival following CVD, IS and PVD for PPIs and H2RAs users by Null, stratified Cox-PH, and Propensity score matched COX-PH models regarding different grace periods.

| **Model** | **Grace period** | **Cardio Vascular Diseases (CVD)^1^** | | **Ischemic Stroke (IS)^2^** | | **Peripheral Vascular Diseases (PVD)^3^** | |
| --- | --- | --- | --- | --- | --- | --- | --- |
|  |  | **Crude HR [95%CI]** | **Adjusted HR [95%CI]** | **Crude HR [95%CI]** | **Adjusted HR [95%CI]** | **Crude HR [95%CI]** | **Adjusted HR [95%CI]** |
| **Unadjusted** | non-grace period | 0.75 [0.64-0.89] | - | 1.34 [0.93-1.95] | - | 4.41 [1.09-17.79] | - |
|  | grace period=30 | 1.78 [1.51-2.10] | - | 4.14 [2.85-6.01] | - | 17.52 [4.34-70.72] | - |
|  | grace period=90 | 1.73 [1.47-2.04] | - | 4.01 [2.77-5.82] | - | 16.25 [4.03-65.61] | - |
|  | grace period=180 | 1.69 [1.43-1.99] | - | 3.89 [2.68-5.65] | - | 16.10 [3.99-64.98] | - |
|  | grace period=360 | 1.63 [1.38-1.92] | - | 3.75 [2.58-5.44] | - | 15.65 [3.88-63.19] | - |
| **On-treatment: Stratified Cox-PH adjusted for baseline covariates*** | non-grace period | - | 0.71 [0.59-0.85] | - | 1.30 [0.89-1.90] | - | 5.05 [1.21-21.12] |
|  | grace period=30 | - | 1.17 [0.95-1.43] | - | 1.97 [1.28-3.05] | - | 6.63 [1.46-30.12] |
|  | grace period=90 | - | 1.15 [0.94-1.42] | - | 1.92 [1.24-2.97] | - | 5.49 [1.22-24.61] |
|  | grace period=180 | - | 1.12 [0.91-1.38] | - | 1.82 [1.17-2.81] | - | 5.54 [1.24-24.79] |
|  | grace period=360 | - | 1.09 [0.89-1.34] | - | 1.78 [1.15-2.75] | - | 5.10 [1.14-22.97] |
| **1:1 Propensity score-matched [PSM]**** | non-grace period | - | 0.76 [0.61-0.95] | - | 1.18 [0.74-1.87] | - | 4.542[1.02-20.15] |
|  | grace period=30 | - | 1.81 [1.45-2.27] | - | 3.65 [2.28-5.84] | - | 18.85 [4.22-84.14] |
|  | grace period=90 | - | 1.76 [1.44-2.20] | - | 3.53 [2.20-5.64] | - | 17.07 [3.82-76.25] |
|  | grace period=180 | - | 1.71 [1.37-2.14] | - | 3.40 [2.13-5.44] | - | 17.07[3.82-76.25] |
|  | grace period=360 | - | 1.66 [1.33-2.08] | - | 3.23 [2.02-5.16] | - | 17.07[3.82-76.25] |

**Notes:** 1* Model: adjusted for alcohol abuse and diastolic blood pressure, stratified by clopidogrel, baseline age (≤60/>60), NSAIDs, steroids, number of hospital visits (≤2,>2), systolic blood pressure(<80/≥80), Charlson's comorbidity index (≤1,>1) index years, and dyslipidemia; 2* Model: adjusted by steroids, clopidogrel, NSAIDs, diastolic blood pressure (<120/≥120), and alcoholic abuse, stratified by baseline age, index years, Charlson's comorbidity index (≤1,>1), dyslipidemia, number of hospital visits (≤2,>2), systolic blood pressure (<80/>=80); 3* Model: adjusted by number of hospital visits(≤2,>2), systolic blood pressure (<80/≥80), steroids, diastolic blood pressure (<120/≥120), and alcoholic abuse, stratified by baseline age(≤60/>60), index years, Charlson's comorbidity index (≤1,>1), dyslipidemia, clopidogrel, NSAIDs; 1-3** Model: Propensity score matching by sex, baseline age (≤60/>60), index years, diabetes, dyslipidemia, rheumatoid arthritis, Charlson's comorbidity index (CCI), NSAID, Clopidogrel, Steroids, Diuretics, number of hospital visits, baseline systolic blood pressure and diastolic blood pressure.


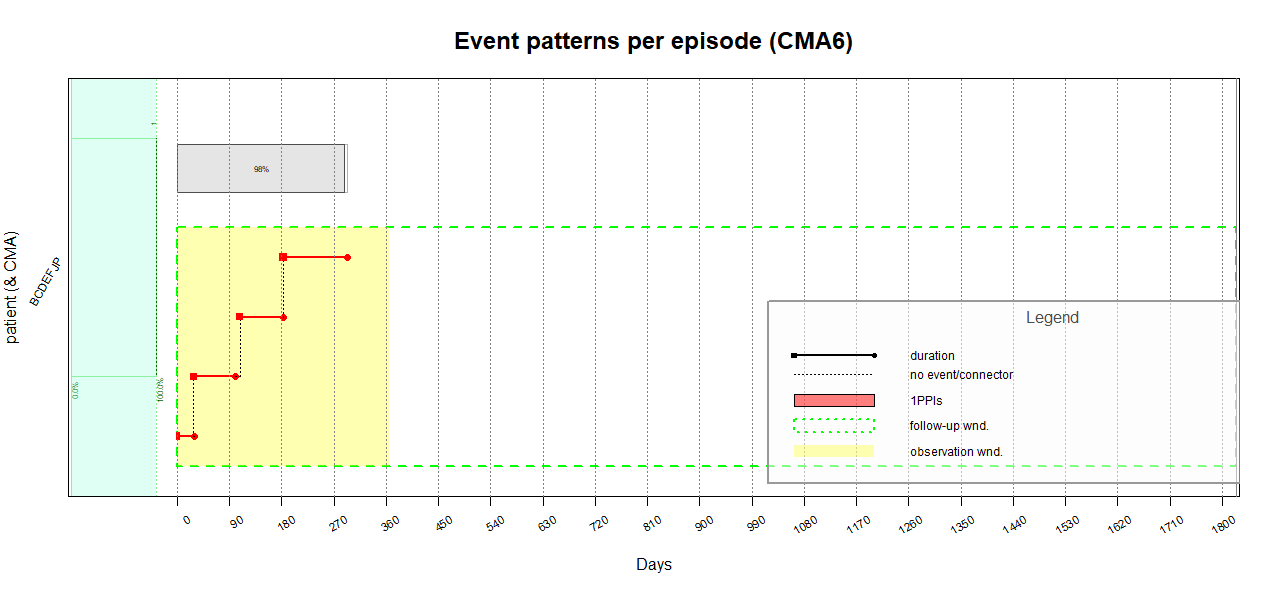


**Figure 4S** Medication Possession Ratio (MPR) measured with continuous multiple-interval measures of medication availability (CMA) ^(Dima & Dediu, 2017)^; CMA6 computed the number of gap days for all event intervals, which were extracted from the total time interval, accounting for carry over within observation window (=365 days) and excluding the medication left. The MPR for patient “BCDEFJP” was 98.0%; the narrower gaps between each medication episode, the higher percentage of medication persistent use.


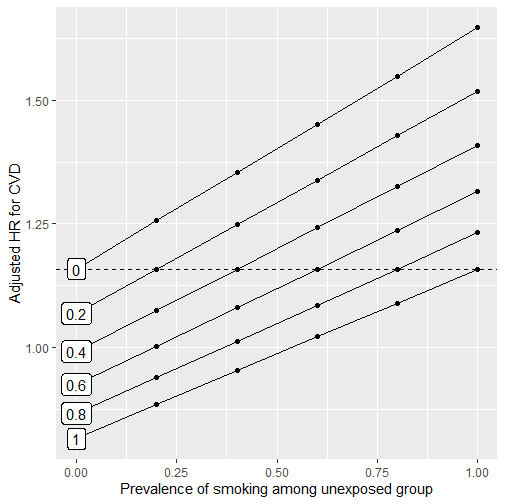

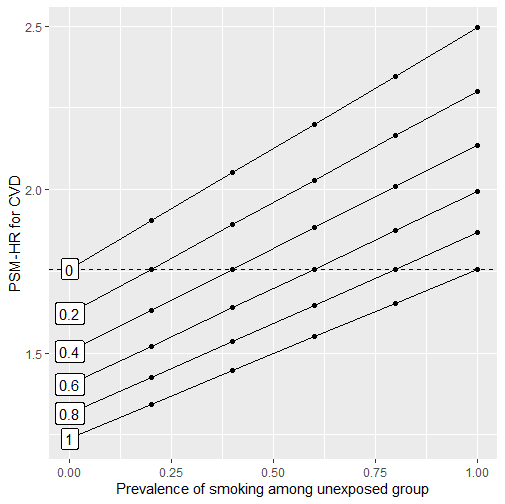


(A) Stratified Cox-proportional hazard model (B) Propensity-score matched model

**Figure 5S** Array-based sensitivity analysis(D’Agostino McGowan, 2022) of a binary unmeasured confounder (smoking) with an assumed relationship with the cardio vascular disease of (HR = 1.42)(Woodward et al., 2005) on an observed hazard ratio of 1.16 (A) and 1.76 (B) (dashed line). The x-axis shows the assumed prevalence of the smoking in the unexposed group; each line represents a different prevalence of smoking in the exposed group. The y-axis shows the corresponding relationship between the exposure and outcome after adjusting for smoking.


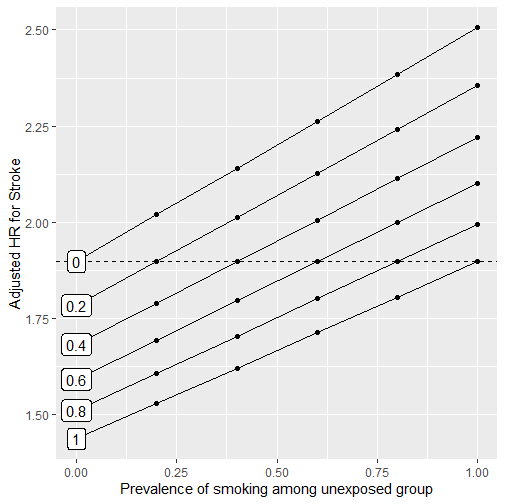

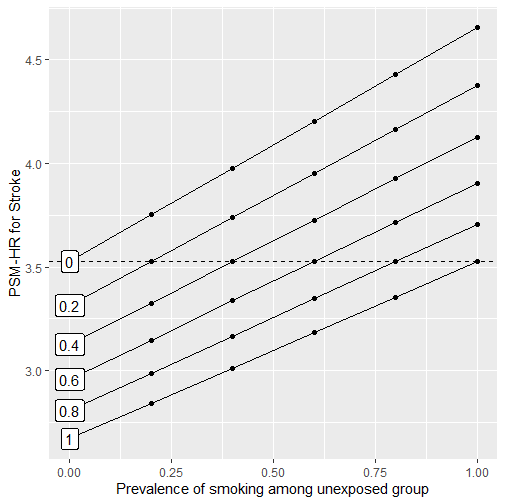


(A) Stratified Cox-proportional hazard model (B) Propensity-score matched model

**Figure 6S** Array-based sensitivity analysis(D’Agostino McGowan, 2022) of a binary unmeasured confounder (smoking) with an assumed relationship with the ischemic stroke of (HR = 1.32)(Woodward et al., 2005) on an observed hazard ratio of 1.92 (A) and 3.53 (B) (dashed line). The x-axis shows the assumed prevalence of smoking in the unexposed group; each line represents a different prevalence of smoking in the exposed group. The y-axis shows the corresponding relationship between the exposure and outcome after adjusting for smoking.


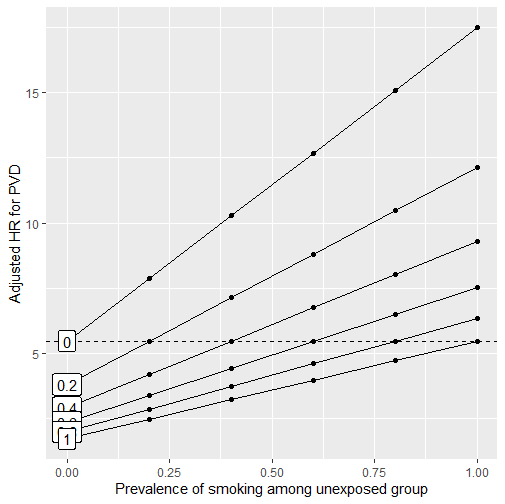

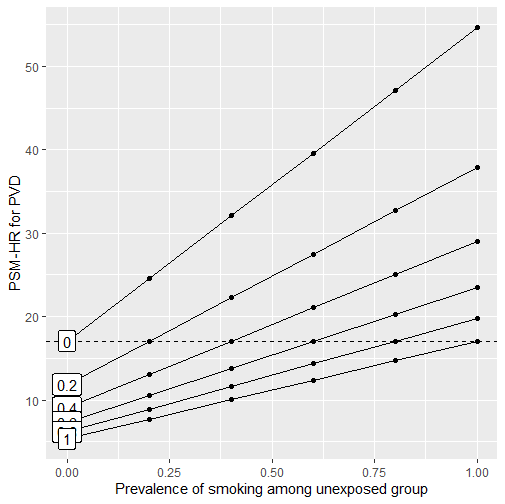


(A) Stratified Cox-proportional hazard model ( B) Propensity-score matched model

**Figure 7S** Array-based sensitivity analysis(D’Agostino McGowan, 2022) of a binary unmeasured confounder (smoking) with an assumed relationship with the peripheral vascular disease of (HR = 3.20)(Ding et al., 2019) on an observed hazard ratio of 5.49 (A) and 17.07 (B) (dashed line). The x-axis shows the assumed prevalence of smoking in the unexposed group; each line represents a different prevalence of smoking in the exposed group. The y-axis shows the corresponding relationship between the exposure and outcome after adjusting for smoking.


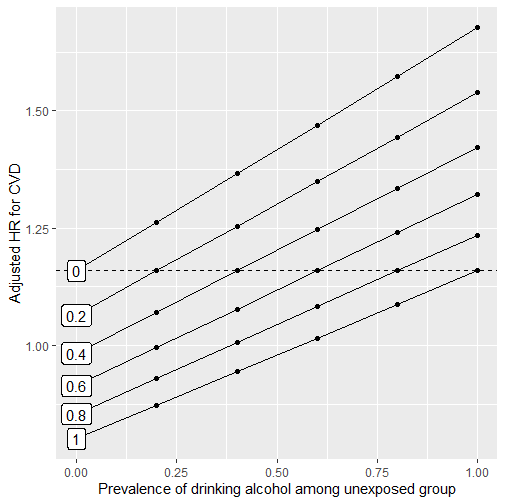

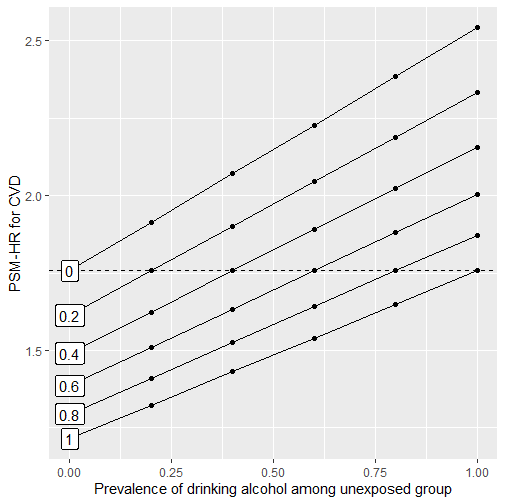


(A) Stratified Cox-proportional hazard model ( B) Propensity-score matched model

**Figure 8S** Array-based sensitivity analysis(D’Agostino McGowan, 2022) of a binary unmeasured confounder (alcohol consumption) with an assumed relationship with the cardio vascular disease of (HR = 1.45)(Sung et al., 2022) on an observed hazard ratio of 1.16 (A) and 1.76 (B) (dashed line). The x-axis shows the assumed prevalence of alcohol consumption in the unexposed group; each line represents a different prevalence of alcohol consumption in the exposed group. The y-axis shows the corresponding relationship between the exposure and outcome after adjusting for alcohol consumption.


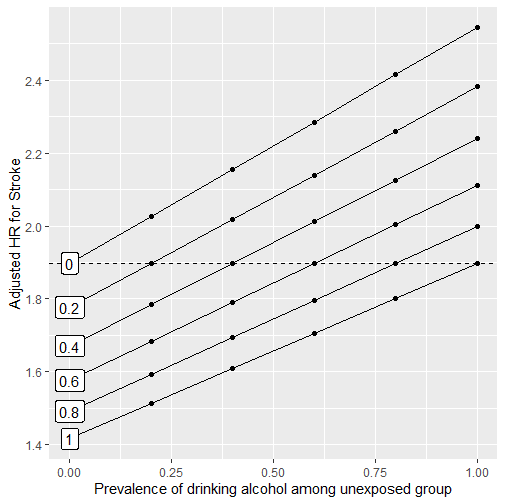

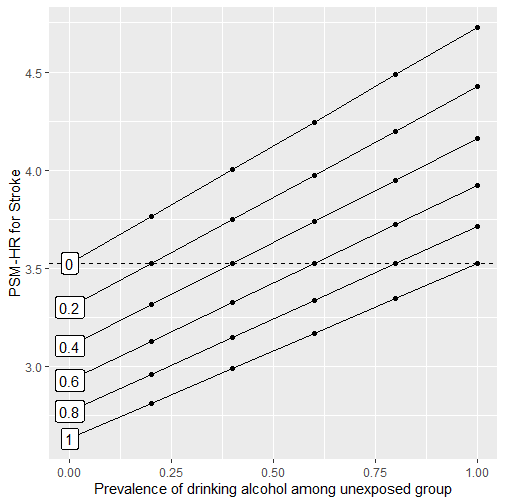


(A) Stratified Cox-proportional hazard model (B) Propensity-score matched model

**Figure 9S** Array-based sensitivity analysis(D’Agostino McGowan, 2022) of a binary unmeasured confounder (alcohol consumption) with an assumed relationship with the ischemic stroke of (HR = 1.34)(Kadlecova, Andel, Mikulik, Handing, & Pedersen, 2015) on an observed hazard ratio of 1.92 (A) and 3.53 (B) (dashed line). The x-axis shows the assumed prevalence of alcohol consumption in the unexposed group; each line represents a different prevalence of alcohol consumption in the exposed group. The y-axis shows the corresponding relationship between the exposure and outcome after adjusting for alcohol consumption.


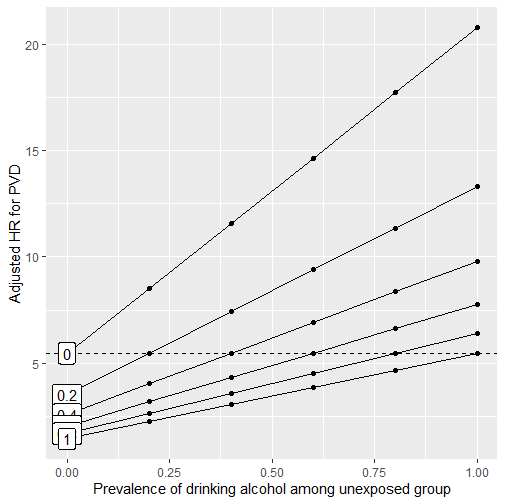

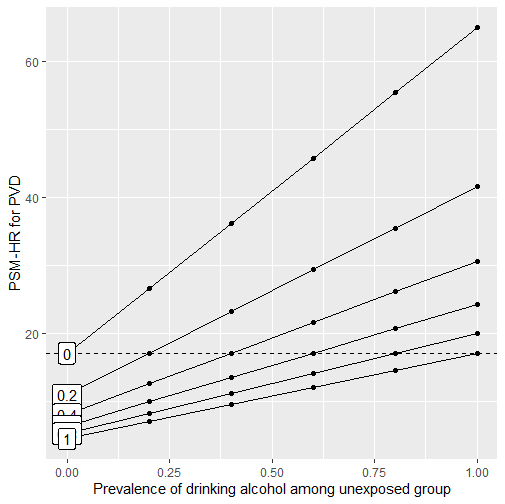


(A) Stratified Cox-proportional hazard model (B) Propensity-score matched model

**Figure 10S** Array-based sensitivity analysis(D’Agostino McGowan, 2022) of a binary unmeasured confounder (alcohol consumption) with an assumed relationship with the peripheral vascular disease of (HR = 3.80)(Huang et al., 2017) on an observed hazard ratio of 5.49 (A) and 17.07 (B) (dashed line). The x-axis shows the assumed prevalence of alcohol consumption in the unexposed group; each line represents a different prevalence of alcohol consumption in the exposed group. The y-axis shows the corresponding relationship between the exposure and outcome after adjusting for alcohol consumption.

**Multiple – bias sensitivity analysis**

To measure potential joint of selection bias, misclassification of exposure, and related unmeasured confounders affecting outcome, sensitivity analysis was adopted via multi-bias E-values. The study sample was of participants in a University hospital that PPIs /H2RAs users could have been affected by the characteristcs of study site, while, there were unadjusted result to unmeasured confunding, such as, physical activities, BMI, long-life risk factors as shown on below figure 11S.

CVD

PPIs

S

X’

U

**Figure 11S** Multiple-bias framework with S = Selection bias (Protopathic bias), U = Unmeasured confounders, PPIs -> X’ = Different misclassification of exposure

Based on the above framework, we assumed that there was misclassification of exposure due to self-treatment of PPIs or H2RAs, whereas, the bias characteristics is common to the entire selected population. In addition, the outcomes were presumed to be rare (less than 15%). We calculated multi-bias E-values for unmeasured confounders but consider multiple biases. *EValue* package in R("Introduction to multiple-bias sensitivity analysis Biases," 2022) was applied to analyze the results.

In table 5S, for instance, with an E-values of 1.15, all sensitivity parameters for each bias would have to take on for an observed hazard ratio (1.76) to be compatible with a null hazard ratio, while, E-values of 1.09 for all sensitivity parameters of each bias would have to move 95%CI of the observed hazard ratio to include the null.

**Table 5S** Multiple-bias sensitivity analysis for CVD, IS, and PVD outcomes with E-values

| **Model** | **Cardio Vascular Diseases (CVD)^1^** | | | | **Ischemic Stroke (IS)^2^** | | | | **Peripheral Vascular Diseases (PVD)^3^** | | | |  |
| --- | --- | --- | --- | --- | --- | --- | --- | --- | --- | --- | --- | --- | --- |
|  | **Crude HR [95%CI]** | **Adjusted HR [95%CI]** | **E-Values (estimate)** | **E-Values (CI)** | **Crude HR [95%CI]** | **Adjusted HR [95%CI]** | **E-Values (estimate)** | **E-Values (CI)** | **Crude HR [95%CI]** | **Adjusted HR**  **[95%CI]** | **E-Values (estimate)** | **E-Values (CI)** | |
| Null | 1.73 [1.47-2.04] | - | - | - | 4.01 [2.77-5.82] | - | - | - | 16.25 [4.03-65.61] | - | - | - | |
| On-treatment: Stratified Cox-PH adjusted for baseline covariates* | - | 1.16 [0.94-1.42] | 1.04 | 1.000 | - | 1.90 [1.27-2.84] | 1.17 | 1.06 | - | 5.47[1.22-24.52] | 1.49 | 1.05 | |
| 1:1 Propensity score-matched [PSM]** | - | 1.76 [1.40-2.20] | 1.15 | 1.09 | - | 3.53 [2.20-5.64] | 1.35 | 1.21 | - | 17.07 [3.82-76.25] | 1.91 | 1.37 | |

**Notes:** 1* Model: adjusted for alcohol abuse, diabetes and diastolic blood pressure, stratified by clopidogrel, baseline age (≤60/>60), NSAIDs, steroids, number of hospital visits (≤2,>2), systolic blood pressure(<80/≥80), Charlson's comorbidity index (≤1,>1) index years, and dyslipidemia; 2* Model: adjusted by clopidogrel, NSAIDs, diastolic blood pressure (<120/≥120), and alcoholic abuse, stratified by baseline age, index years, Charlson's comorbidity index (≤1,>1), dyslipidemia, diabetes, steroids, number of hospital visits (≤2,>2), systolic blood pressure (<80/>=80); 3* Model: adjusted by number of hospital visits(≤2,>2), systolic blood pressure (<80/≥80), steroids, diastolic blood pressure (<120/≥120), and alcoholic abuse, stratified by baseline age(≤60/>60), index years, Charlson's comorbidity index (≤1,>1), dyslipidemia, diabstes, clopidogrel, NSAIDs; 1-3** Model (n=2254;2254;7856): Propensity score matching by sex, baseline age (≤60/>60), index years, diabetes, dyslipidemia, rheumatoid arthritis, Charlson's comorbidity index (CCI), NSAID, Clopidogrel, Steroids, Diuretics, number of hospital visits, baseline systolic blood pressure and diastolic blood pressure.

**Description of Study Hospital (Laohaviriyakamol, 2016)**

Songklanagarind Hospital is a university teaching hospital, affiliated to the Faculty of Medicine, Prince of Songkla University, located in the South of Thailand. It is the first university hospital in Southern Thailand, as well as, being a super tertiary care institution with 1,000 patient’s beds. Being a university teaching hospital, it comprises of medical specialty services in which the hospital has the professional staff who provide excellent services to complicated medical conditions. Most patients are university students and staffs, as well as, Thai citizen who are referred from the other public hospitals in the south of Thailand. The number of patients who visit hospital were over 1 million per year.

Asides from out-/inpatient and emergency services, there are six medical centers established to serve citizen in the South of Thailand as following:

- Naradhiwas Rajanagarindra Heart Center
- Nanthana-Kriangkrai Chotiwattanaphan (NKC) Center of Gastroenterology and Hepatology
- Cancer Center
- Trauma Center
- Medical Information Center
- Palliative Care Unit

**References**

D’Agostino McGowan, L. (2022). Sensitivity Analyses for Unmeasured Confounders. *Current Epidemiology Reports*, 15. doi:10.1007/s40471-022-00308-6

Dima, A. L., & Dediu, D. (2017). Computation of adherence to medication and visualization of medication histories in R with AdhereR: Towards transparent and reproducible use of electronic healthcare data. *PLoS One, 12*(4), e0174426. doi:10.1371/journal.pone.0174426

Ding, N., Sang, Y., Chen, J., Ballew, S. H., Kalbaugh, C. A., Salameh, M. J., . . . Matsushita, K. (2019). Cigarette Smoking, Smoking Cessation, and Long-Term Risk of 3 Major Atherosclerotic Diseases. *J Am Coll Cardiol, 74*(4), 498-507. doi:10.1016/j.jacc.2019.05.049

Huang, J.-Y., Chen, W.-K., Lin, C.-L., Lai, C.-Y., Kao, C.-H., & Yang, T.-Y. (2017). Increased risk of peripheral arterial disease in patients with alcohol intoxication: A population-based retrospective cohort study. *Alcohol, 65*, 25-30. doi:<https://doi.org/10.1016/j.alcohol.2017.06.003>

Introduction to multiple-bias sensitivity analysis Biases. (2022). Retrieved from <https://cran.r-project.org/web/packages/EValue/vignettes/multiple-bias.html>

Kadlecova, P., Andel, R., Mikulik, R., Handing, E. P., & Pedersen, N. L. (2015). Alcohol consumption at midlife and risk of stroke during 43 years of follow-up: cohort and twin analyses. *Stroke, 46*(3), 627-633. doi:10.1161/STROKEAHA.114.006724

Laohaviriyakamol, S. (2016). *Annual Report of Songklanagarind Hospital*. Retrieved from Songkhla:

Rattanaumpawan, P., Wongkamhla, T., & Thamlikitkul, V. (2016). Accuracy of ICD-10 Coding System for Identifying Comorbidities and Infectious Conditions Using Data from a Thai University Hospital Administrative Database. *J Med Assoc Thai, 99*(4), 368-373.

Sung, C., Chung, C. H., Lin, F. H., Chien, W. C., Sun, C. A., Tsao, C. H., & Weng, C. E. (2022). Risk of cardiovascular disease in patients with alcohol use disorder: A population-based retrospective cohort study. *PLoS One, 17*(10), e0276690. doi:10.1371/journal.pone.0276690

Woodward, M., Lam, T. H., Barzi, F., Patel, A., Gu, D., Rodgers, A., . . . Asia Pacific Cohort Studies, C. (2005). Smoking, quitting, and the risk of cardiovascular disease among women and men in the Asia-Pacific region. *Int J Epidemiol, 34*(5), 1036-1045. doi:10.1093/ije/dyi104

Zhao, Q. Y., Luo, J. C., Su, Y., Zhang, Y. J., Tu, G. W., & Luo, Z. (2021). Propensity score matching with R: conventional methods and new features. *Ann Transl Med, 9*(9), 812. doi:10.21037/atm-20-3998
